# Role of endoplasmic reticulum stress-mediated autophagy in cadmium-induced apoptosis in BRL-3A cells

**DOI:** 10.3389/fvets.2026.1939951

**Published:** 2026-08-26

**Authors:** Junbing Mao, Hao Ling, Bing Xu, Yaning Shi, Yue Wang, Huimin Chen, Jicang Wang

**Affiliations:** College of Animal Science and Technology, Henan University of Science and Technology, Luoyang, Henan, China

**Keywords:** apoptosis, autophagy, BRL-3A cells, cadmium, endoplasmic reticulum stress, ER-phagy

## Abstract

Cadmium (Cd), a widely present environmental toxicant, can cause liver damage following chronic exposure. Although endoplasmic reticulum stress (ERS) and autophagy are known to be involved in Cd-induced liver injury, their molecular mechanisms remain incompletely understood. In this study, using an *in vitro* Cd exposure model and pharmacological interventions, we investigated the mechanisms through which Cd induces autophagy via ERS and its interplay with apoptosis. We found that Cd-induced autophagosome accumulation was linked to the activation of ERS pathways (PERK/eIF2α/ATF4, IRE1α/JNK/Beclin1, and ATF6). Furthermore, this accumulation was also attributable to Cd-induced impairment of autophagic degradation, as evidenced by p62 accumulation. In addition, Cd exposure activates FAM134B-dependent ER-phagy, which requires ERS regulation. Finally, the autophagy activator rapamycin alleviated apoptosis, whereas the inhibitor chloroquine exacerbated it. In summary, our findings reveal a mechanism through which Cd coordinately regulates hepatocyte fate via ER stress-mediated, FAM134B-dependent ER-phagy and canonical autophagy, offering novel therapeutic targets and theoretical foundations for mitigating Cd toxicity.

## Graphical abstract

**Figure:**
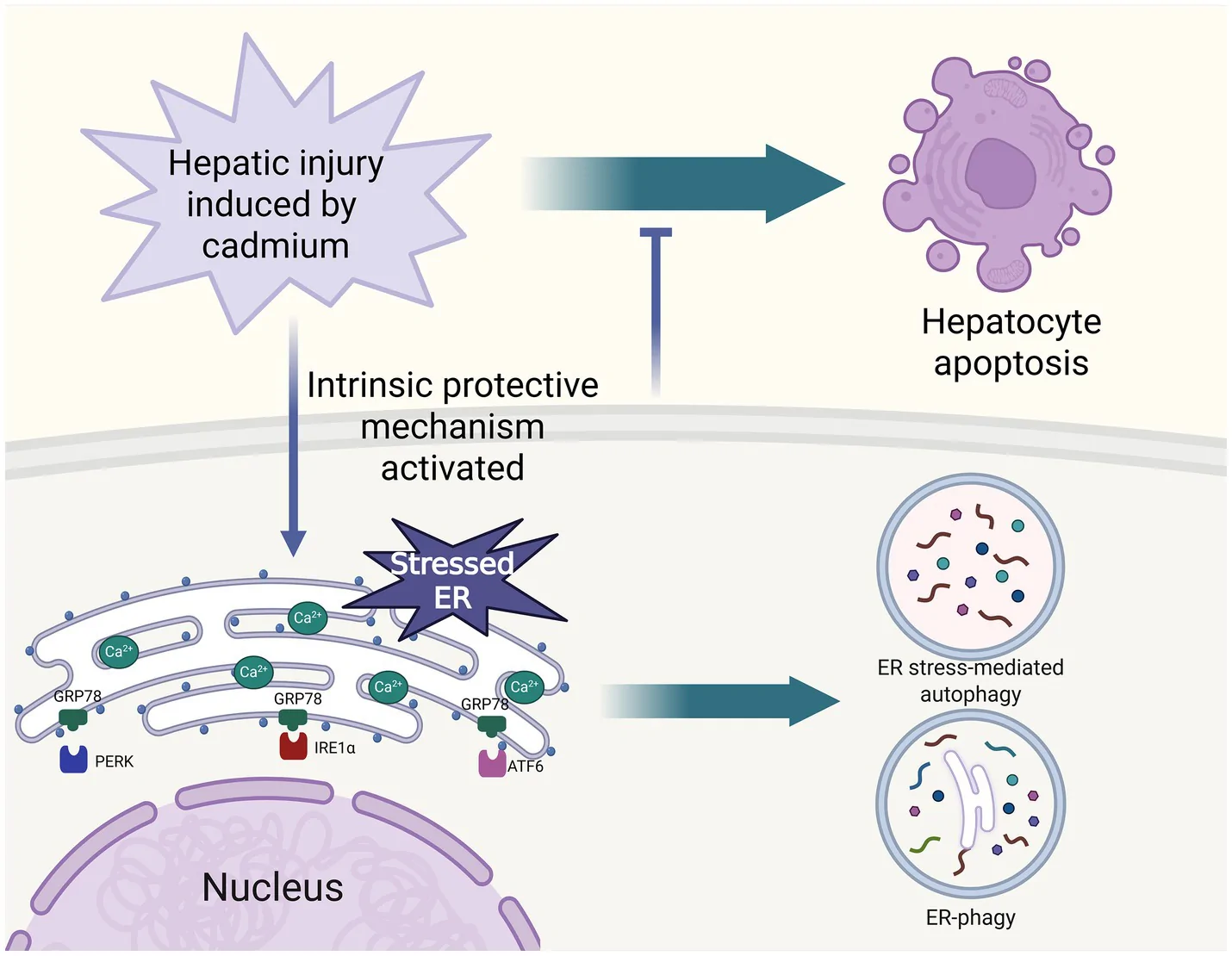

## Highlights

- Cd can induce ERS and lead to increased autophagy and apoptosis.
- Cd-induced accumulation of autophagosomes results from both ERS-mediated initiation and impaired lysosomal degradation.
- Cd activates three ERS signaling axes (PERK/eIF2α/ATF4, IRE1α/JNK/Beclin1, ATF6) to drive hepatocyte autophagy.
- Cd-induced ER-phagy mediated by FAM134B requires regulation through ERS.
- ER stress-mediated autophagy protects against Cd-induced apoptosis, thereby promoting cell survival.

## 1 Introduction

Cadmium (Cd) is a prevalent toxic heavy metal, historically utilized in painting and plating processes, and currently employed in semiconductors, environmental monitoring, and bio-imaging (1, 2). Additionally, cigarette smoke and batteries are among the primary sources of Cd exposure (3, 4). Research has shown a marked increase in serum and urinary Cd levels in smokers, with a significantly higher incidence of bone pain compared to non-smokers (5). Cd enters the body through ingestion or inhalation and accumulates in various organs, including the liver, kidneys, and testes. The liver, in particular, is considered a major target organ and is highly susceptible to Cd-induced damage (6, 7). Numerous studies have identified a positive correlation between Cd exposure and a range of liver diseases, such as necrotizing inflammation, hyperglycemia, nonalcoholic fatty liver disease, and nonalcoholic steatohepatitis (8, 9). *In vivo* experiments have demonstrated that Cd exposure leads to histological alterations, structural degradation of liver tissue, and a significant rise in inflammatory factors and oxidative damage markers (10). *In vitro* studies reveal that Cd exposure increases reactive oxygen species (ROS) production, disrupts antioxidant enzyme expression and activity, damages essential metals and cellular thiol groups, modulates autophagy and apoptosis, and inhibits DNA repair enzymes (11–13). However, the precise intracellular mechanisms, particularly those involving organelle stress, remain incompletely understood.

The endoplasmic reticulum (ER) serves as the primary site for protein synthesis and maturation, and is crucial for hepatic metabolism and detoxification. Consequently, impairment of ER function can directly compromise liver functionality and potentially trigger a cascade of diseases (14, 15). Studies have indicated that the ER is a major target of Cd toxicity. When disrupted by adverse factors, the ER undergoes homeostatic changes, resulting in the accumulation of unfolded or misfolded proteins and the induction of endoplasmic reticulum stress (ERS) (16, 17). In response, the unfolded protein response (UPR) is activated to promote autophagy, degrade misfolded proteins, and reduce new protein synthesis, thereby restoring ER homeostasis (18, 19). However, if the UPR fails to adequately resolve ERS, it can lead to apoptosis (20, 21). Thus, ERS plays a pivotal role in regulating both cellular autophagy and apoptosis. Research has demonstrated that ERS mediates autophagy in Cd-exposed renal tubular epithelial cells (22). Similarly, experiments have shown that ER stress-mediated autophagy activation contributes to hepatotoxic injury in Cd-exposed ducks *in vivo* (23). Nevertheless, the *in vitro* mechanisms by which ERS mediates Cd-induced autophagy in hepatocytes remain incompletely understood. Furthermore, the specific role of ER stress-mediated autophagy in Cd-induced hepatocyte apoptosis has yet to be clarified.

Endoplasmic reticulum autophagy (ER-phagy), a form of selective autophagy, mediates the degradation of excessively swollen ER caused by the accumulation of misfolded/unfolded proteins, alleviating ERS to maintain ER volume and homeostasis (24). FAM134B (also known as RETREG1, a reticulophagy regulator 1) is the first mammalian ER-phagy receptor discovered. It recruits autophagosomes by binding to LC3, thereby promoting the engulfment and degradation of ER fragments (25). Studies have shown that CdTe quantum dots can induce both ERS and ER-phagy in renal cells (26). However, it remains unclear whether Cd-induced, ER stress-mediated autophagy involves FAM134B-dependent ER-phagy in hepatocytes, and the functional roles and underlying mechanisms of this process have yet to be elucidated.

Based on the aforementioned background, elucidating the roles of ER stress-mediated autophagy and ER-phagy in Cd-induced hepatocyte apoptosis is essential. While our previous work has established cadmium hepatotoxicity and its correlation with ER stress and apoptosis, the present study specifically investigates the function of ER stress-driven autophagy and ER-phagy in this process. Using the BRL-3A cell line as an *in vitro* model, we explored the mechanisms through which ER stress pathways activate autophagy. Furthermore, pharmacological inhibitors and activators were used to dissect the functional interplay between apoptosis and autophagy. This study offers novel theoretical insights into the molecular mechanisms underlying cadmium-induced liver injury and suggests potential avenues for therapeutic intervention.

## 2 Materials and methods

### 2.1 Reagents

DMEM high-glucose medium (G4510–500 mL) was acquired from Servicebio Biotechnology Co., Ltd. (Wuhan, China). Fetal bovine serum (FBS; 03. U16001-500 mL) was sourced from EallBio (Beijing, China). Penicillin–streptomycin liquid (C0222) and Trypsin Solution (C0205) were purchased from Beyotime Biotechnology Co., Ltd. (Shanghai, China). Cd chloride (CdCl₂, 99.99%, 10,108-64-2) and 4-phenylbutyric acid (4-PBA, 99%, 1821-12-1) were purchased from Sigma-Aldrich (Shanghai, China). Chloroquine diphosphate (CQ, C6628) and Rapamycin (Rap, V900930) were procured from Macklin Biochemical Technology Co., Ltd. (Shanghai, China).

### 2.2 Cell culture and treatment

The rat hepatocyte cell line BRL-3A was obtained from the College of Veterinary Medicine, Yangzhou University (Jiangsu, China). Cells were cultured in DMEM high-glucose medium supplemented with 10% FBS, 100 U/mL penicillin, and 100 U/mL streptomycin, and maintained at 37 °C in a 5% CO_2_. To establish the optimal Cd exposure parameters for autophagy studies, cells were exposed to CdCl₂ at concentrations of 0, 3, 6, 12, 24, 48, and 96 μM for 12, 24, or 48 h. A concentration range of 0–12 μM CdCl₂ with 24-h exposure was selected for subsequent analyses of ERS, autophagy, and apoptosis. To investigate ERS-autophagy crosstalk, cells were co-treated with 6 μM CdCl₂ and either: 10 mM 4-phenylbutyric acid (4-PBA, ERS inhibitor), 40 μM chloroquine (CQ, autophagy inhibitor), and 60 nM rapamycin (Rap, autophagy activator). For mechanistic studies on ER stress-mediated autophagy, cells were exposed to 0–12 μM CdCl₂ for 24 h. To delineate the relationship between ERS and ER-phagy, an additional experimental group received 6 μM CdCl₂ combined with 10 mM 4-PBA. The autophagy-apoptosis interplay was assessed using 6 μM CdCl₂ co-administered with 40 μM CQ or 60 nM Rap. The concentration screening for 4-PBA, CQ, and Rap is described in the Supplementary materials.

### 2.3 Cell viability assay

Cell viability was assessed with the Cell Counting Kit-8 (CCK-8; BS350B, Biosharp, China). Briefly, BRL-3A cells were seeded in 96-well plates and allowed to adhere for 12 h. After processing, 10 μL CCK-8 reagent was added to each well. Following gentle mixing and incubation for 2 h at 37 °C, the absorbance was measured at 450 nm using a full-spectrum microplate reader (BioTek Synergy H1).

### 2.4 Autophagosomes observation by transmission electron microscopy

Ultrastructural analysis of autophagosomes was performed using transmission electron microscopy (TEM). Cd-treated cells were fixed with 2.5% glutaraldehyde and 2.5% paraformaldehyde in 0.1 M phosphate buffer (pH 7.4) for 4 h at 4 °C. After dehydration through a graded ethanol series (50–100%) and acetone substitution, samples were embedded in EPON-812 resin, ultrathin-sectioned (70 nm), and double-stained with uranyl acetate and lead citrate. Autophagic vesicles were visualized using a JEM-1400Plus TEM (JEOL Ltd., Tokyo, Japan) operated at 80 kV.

### 2.5 Autophagic detection via MDC staining

Autophagic vacuoles were detected using a monodansylcadaverine (MDC) staining kit (G0170, Solarbio, China). Following 24-h Cd exposure, cells were washed twice with PBS (pH 7.4) and incubated with MDC working solution (10 μg/mL) at 25 °C for 30 min in the dark. After removing excess stain through three PBS washes, fluorescent images were acquired using an inverted fluorescence microscope (Nikon Eclipse Ti2, Japan) with 380 nm excitation/525 nm emission filters.

### 2.6 Real-time fluorescence quantitative PCR (RT-qPCR)

Gene-specific primers were designed using Primer Premier 5.0 based on conserved sequences from the NCBI database. Total RNA was isolated from hepatocytes using TRIzol reagent (P118, GenStar, China) following cell lysis and ice-cold homogenization. RNA purity and concentration were quantified using a NanoDrop 2000 spectrophotometer (Thermo Fisher Scientific), with samples exhibiting A260/A280 ratios between 1.8 and 2.0 considered suitable for downstream processing. First-strand cDNA synthesis was performed with 1 μg total RNA using the PrimeScript™ RT Reagent Kit (A230, GenStar, China) according to the manufacturer’s protocols. Quantitative PCR amplification was carried out using TB Green™ Fast qPCR Mix (A303, GenStar, China) on a QuantStudio 5 Real-Time PCR System (Bio-Rad, USA), with triplicate technical replicates per sample. Relative gene expression levels were normalized to *β*-actin and calculated using the 2^-∆∆CT^ method. The primer sequences used in the experiment are shown in Supplementary Table S1.

### 2.7 Western blot assay

Cells were lysed with RIPA buffer (P1010, Servicebio, Wuhan) containing protease inhibitors (C2002, Beyotime, Shanghai). Lysates were centrifuged at 12,000 r/min for 15 min at 4 °C, and protein concentrations were determined using the BCA Protein Assay Kit (P0012-1, Beyotime, Shanghai). Equal amounts of protein (30 μg/lane) were denatured in Laemmli buffer by boiling at 95 °C for 15 min. Protein gel electrophoresis was performed, and then the protein was transferred to PVDF membranes. Membranes were blocked with 5% non-fat milk in TBST for 1 h at room temperature, then incubated with primary antibodies (Supplementary Table S2) overnight at 4 °C. After three washes with TBST, membranes were probed with HRP-conjugated secondary antibodies (1:5000) for 1 h at 25 °C. Protein bands were visualized using BeyoECL Star (P0018AM, Beyotime, China) on a ChemiDoc Imaging System (Bio-Rad, USA), with band intensities quantified using ImageJ. The dilution ratios of antibodies used in the experiment are shown in Supplementary Table S2.

### 2.8 Statistical analysis

All statistical analyses were performed using SPSS 26.0. Between-group comparisons were conducted via one-way ANOVA followed by least significant difference *post-hoc* tests. Results are expressed as mean ± SEM. Statistical significance was defined as follows: *p* < 0.05 (significant), and *p* < 0.01 (highly significant).

## 3 Results

### 3.1 Screening of Cd exposure time and concentration

**Figure 1:**
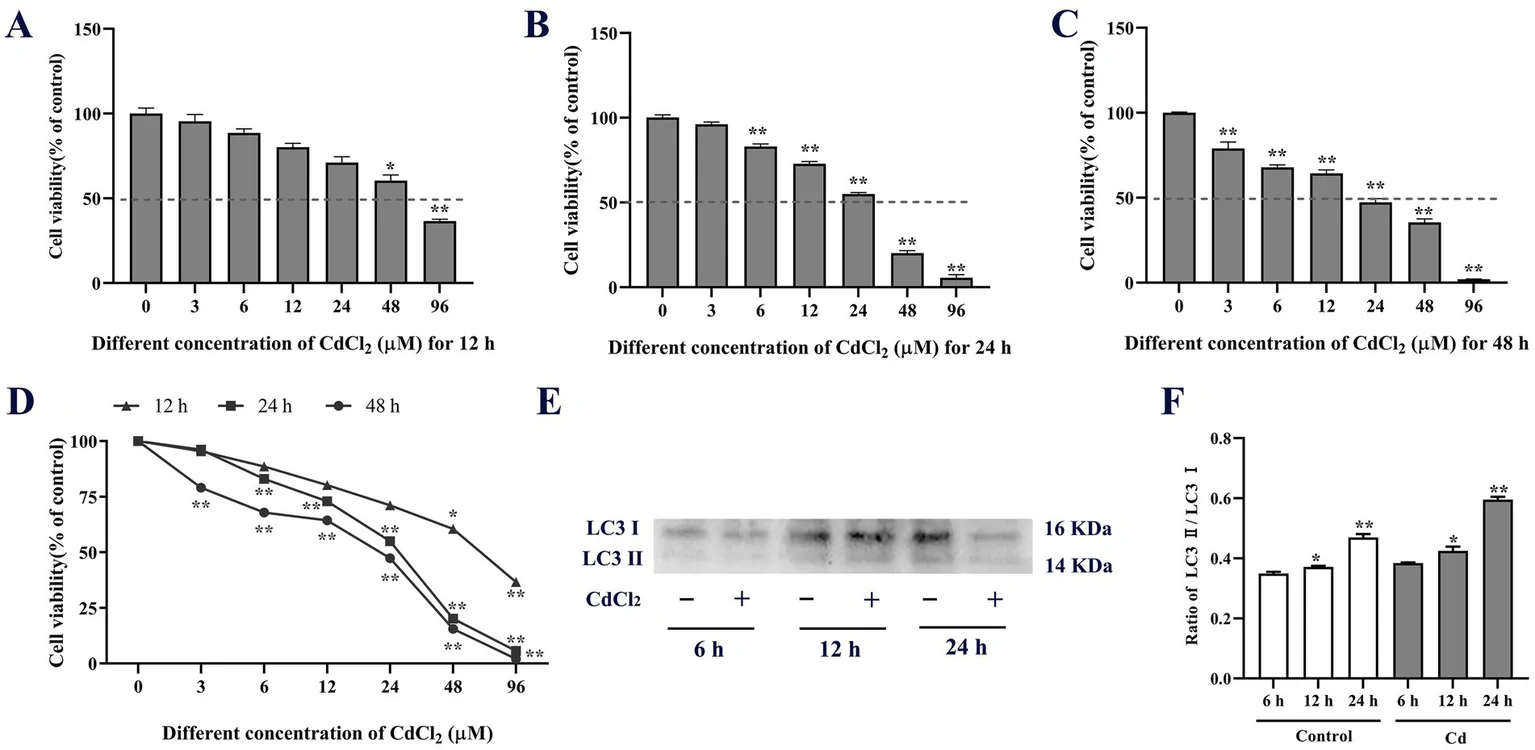
Effects of different concentrations of CdCl_2_ on cell viability at different times. **(A–C)** Effects of different concentrations of Cd on cell viability for 12, 24, and 48 h; **(D)** Line graphs showing cell viability after 12, 24, and 48 h Cd treatment; **(E)** Western blot of LC3; **(F)** Statistical chart of LC3 expression. The data are plotted using mean ± SEM, *n* = 6; **p < 0.01,*p < 0.05 indicate a significant difference compared to the control group.

First, we measured the viability of BRL-3A cells after 12, 24, and 48 h treatment with different concentrations of CdCl_2_ (Figures 1A–D). Progressive reduction in hepatocyte viability was observed with increasing Cd concentrations and extended exposure periods. At 12 h, significant differences emerged between the control group and the 48 μM group (*p* < 0.05), while the 96 μM group showed a significant difference (*p* < 0.01). By 24 h, all treated groups (≥6 μM) exhibited highly significant viability loss compared to the control group (*p* < 0.01). There were significant differences between all Cd treatment groups and the control group after 48 h exposure (*p* < 0.01).

Subsequently, the LC3II/LC3I ratio of autophagosome formation was detected after Cd treatment for 6, 12, and 24 h (Figures 1E,F). The results showed a significant increase in the LC3II/LC3I ratio in the Cd treatment group (6 μM) compared to the control group at 24 h (*p* < 0.01). Based on these results, 6 μM CdCl_2_ with 24 h exposure was selected as the optimal treatment paradigm for subsequent mechanistic investigations.

### 3.2 Effects of Cd exposure on endoplasmic reticulum stress and autophagy in BRL-3A cells

**Figure 2:**
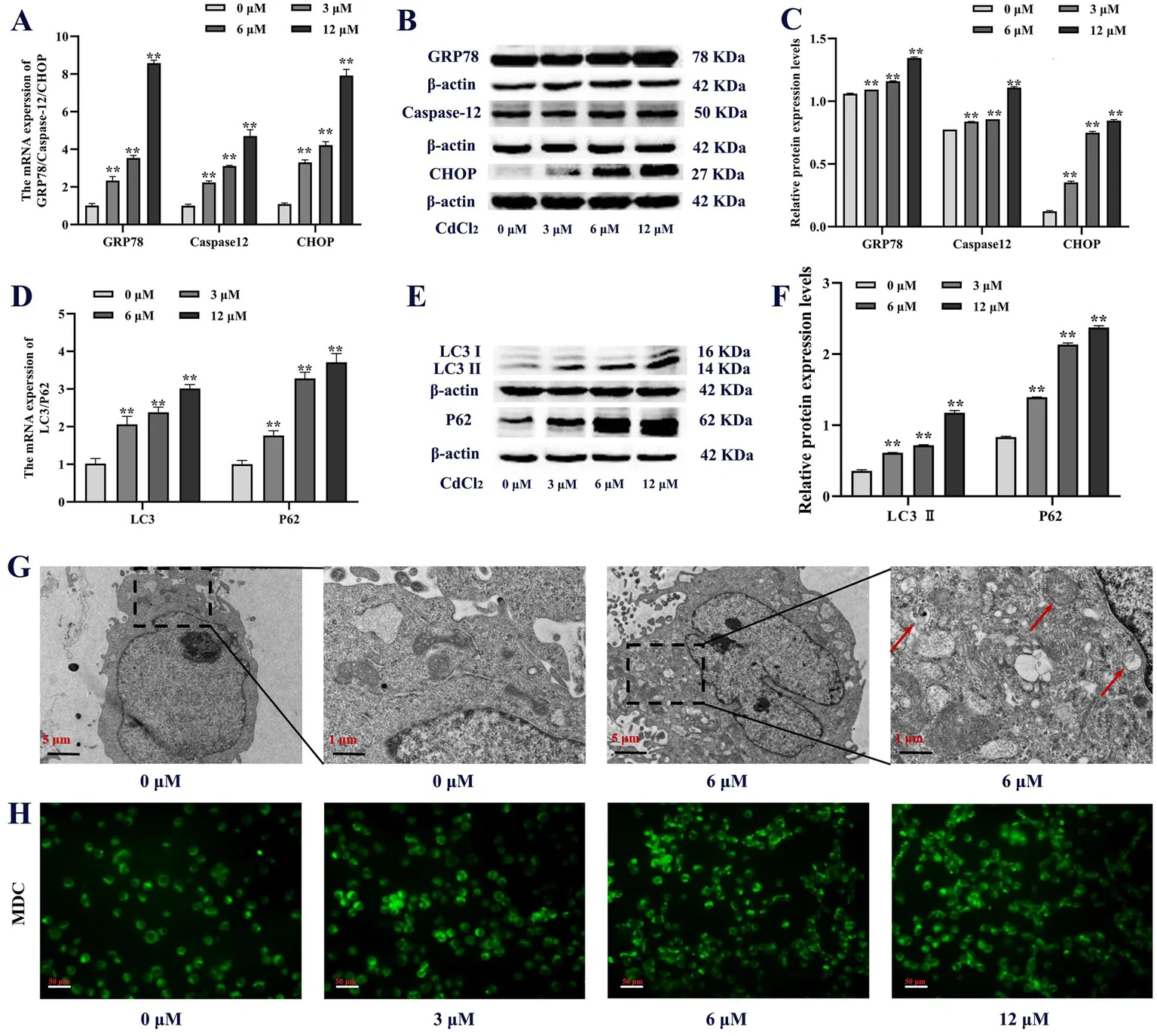
Effects of Cd on ERS and autophagy in BRL-3A cells. **(A)** mRNA expression of ERS markers; **(B)** Western blot of ERS markers; **(C)** Statistical chart of ERS markers expression; **(D)** mRNA expression of autophagy indicators; **(E)** Western blot of autophagy indicators; **(F)** Statistical chart of autophagy indicators expression; **(G)** Observation of autophagosomes by transmission electron microscopy; the red arrows refer to autophagosomes or autolysosomal, 5 μm/1 μm. **(H)** MDC assay for the detection of autophagy; cells undergoing autophagy exhibit bright fluorescent dots distributed in the cytoplasm, 50 μm. the data are plotted using mean ± SEM, *n* = 6; **p < 0.01,*p < 0.05 indicate a significant difference compared to the control group.

Systematic analysis of 0–12 μM CdCl_2_ exposure in BRL-3A hepatocytes revealed concurrent activation of ERS and autophagy. RT-qPCR results demonstrated significant upregulation of ERS markers (GRP78, Caspase-12, CHOP) and autophagy-related genes (LC3, P62) in Cd-treated groups compared to the control (*p* < 0.01) (Figures 2A,D). Parallel immunoblot analysis confirmed the activation of ERS and autophagy at the protein level (Figures 2B,C,E,F). ERS markers and autophagic indicators showed progressive elevation corresponding to Cd concentrations (*p* < 0.01).

Transmission electron microscopy analysis of 6 μM CdCl₂-treated cells revealed substantial accumulation of autophagic vesicles, predominantly double-membrane autophagosomes and autolysosomes (Figure 2G). Complementary MDC fluorescence staining quantified a Cd concentration-dependent increase in autophagic cell populations (Figure 2H). These results demonstrate that Cd exposure activates ERS and induces autophagic responses in BRL-3A cells.

### 3.3 Effect of activation or inhibition of autophagy on autophagic flux under Cd exposure

**Figure 3:**
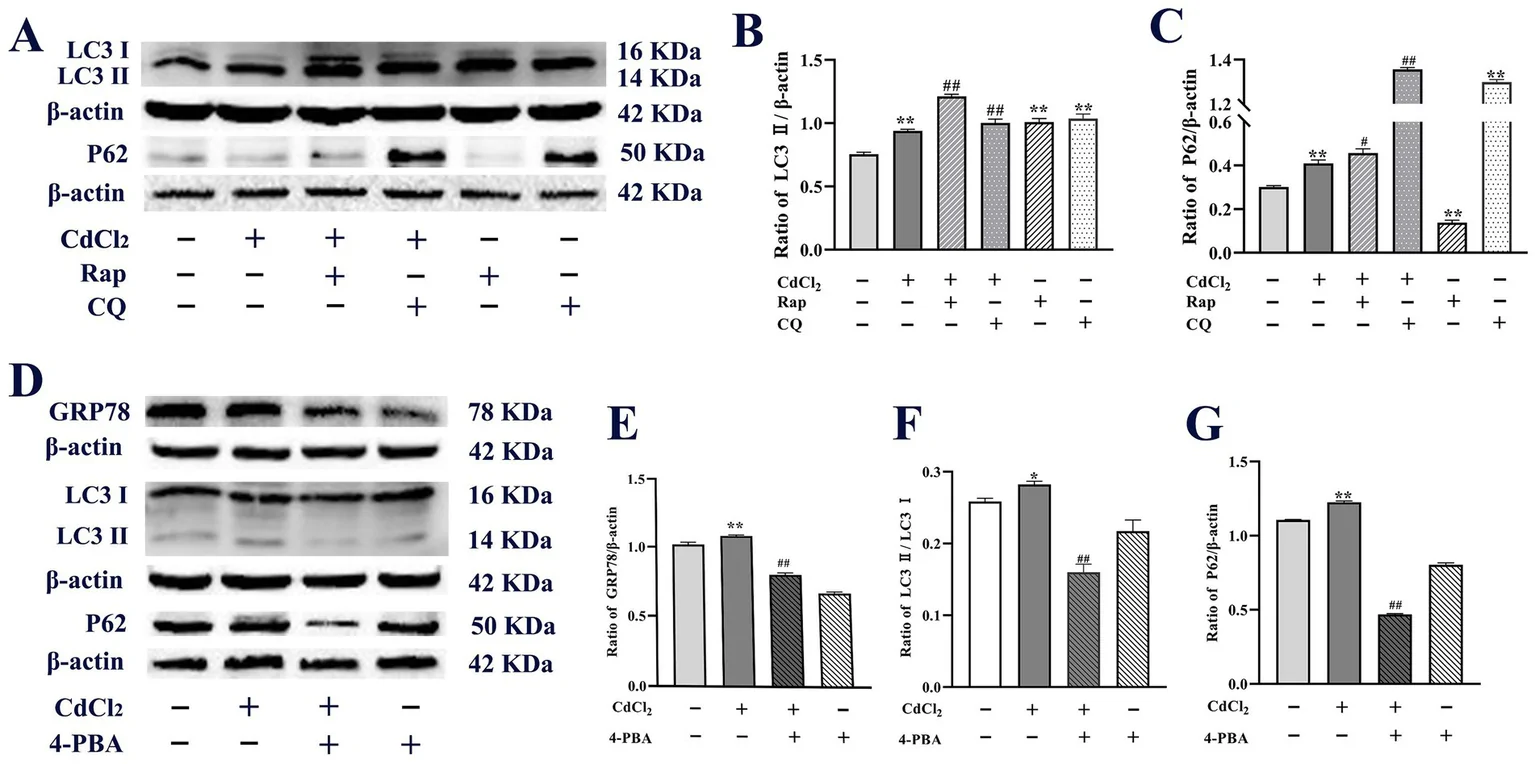
Effects of autophagy modulation (activation/inhibition) and ERS inhibition on autophagic flux and autophagy under Cd exposure. **(A)** Western blot of LC3 and P62 after activation or inhibition of autophagy; **(B,C)** Statistical chart of LC3 II and P62 protein expression after activation or inhibition of autophagy; **(D)** Western blot of GRP78, LC3 and P62 after inhibition of ERS; **(E,F,G)** Statistical chart of GRP78, LC3 II/LC3 I and P62 expression after inhibition of ERS. The data are plotted using mean ± SEM, *n* = 6; **p < 0.01, *p < 0.05 indicate a significant difference compared to the control group. ^##^*p* < 0.01, ^#^*p* < 0.05 indicate a significant difference compared to the Cd group.

Pharmacological modulation using Rap to enhance autophagy and CQ to suppress autolysosomal degradation (Figures 3A–C) revealed distinct autophagic responses. Compared to the control, Cd-treated (6 μM) cells exhibited significantly elevated LC3II and P62 levels (*p* < 0.01), indicating autophagosome accumulation with impaired substrate degradation and disrupted autophagic flux. Notably, both Cd + Rap and Cd + CQ co-treatments further increased LC3II and P62 accumulation relative to Cd-only exposure (*p* < 0.01). Collectively, these findings demonstrate that Cd exposure simultaneously induces autophagic flux and compromises degradative capacity, leading to the marked accumulation of autophagosomes.

### 3.4 Effect of inhibition of endoplasmic reticulum stress on Cd-induced autophagy

While Cd exposure activated both ERS and autophagy, their causal relationship remained undefined. Therefore, on the basis of Cd treatment, we applied 4-PBA intervention in BRL-3A cells to detect the expression of autophagy marker protein (Figures 3D–G). Treatment with 4-PBA significantly reduced the levels of GRP78, the LC3II/LC3I ratio, and P62 compared to the Cd-only group (*p* < 0.01), indicating that ERS was inhibited after the addition of 4-PBA, and Cd-induced autophagic responses depended on ERS.

### 3.5 Molecular mechanisms by which Cd-induced endoplasmic reticulum stress mediates autophagic activation

**Figure 4:**
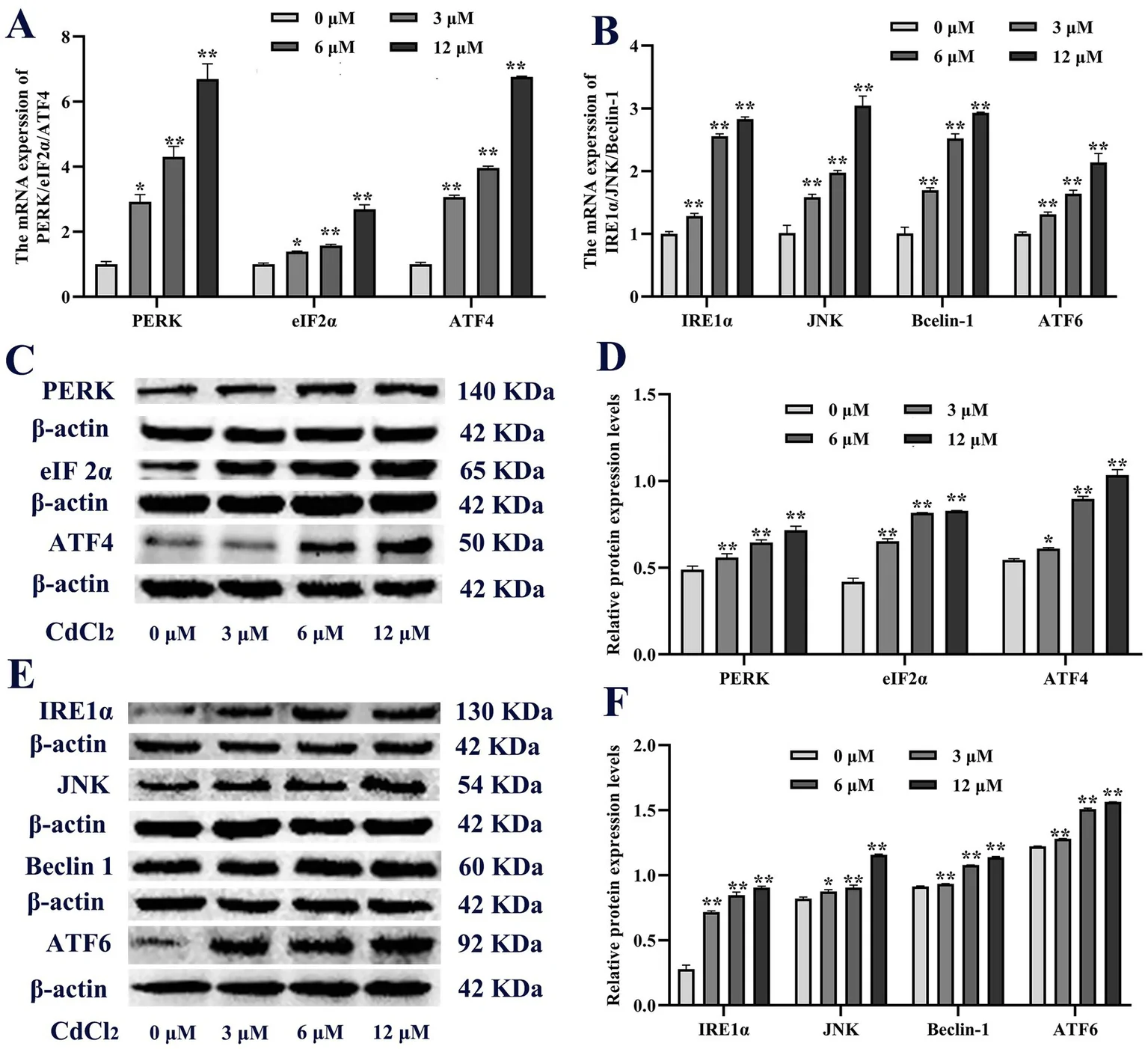
Cd induces protein expression of ER stress-mediated genes associated with the autophagy signaling pathway. **(A)** mRNA expression of PERK, eIF2α, and ATF4. **(B)** mRNA expression of IRE1α, JNK, Beclin1, and ATF6. **(C)** Western blot of PERK, eIF2α, and ATF4. **(D)** Statistical chart of PERK, eIF2α, and ATF4 expression. **(E)** Western blot of IRE1α, JNK, Beclin1, and ATF6. **(F)** Statistical chart of IRE1α, JNK, Beclin1, and ATF6 expression. The data are plotted using mean ± SEM, *n* = 6. **p < 0.01 *p < 0.05 Indicate a significant difference compared to the control group.

To explore the molecular mechanism of Cd-induced ERS mediating autophagy activation in hepatocytes, we detected the mRNA and protein expression levels of related signaling pathway genes by RT-qPCR and Western blot (Figure 4). The results showed that the mRNA and protein levels of key components in the ER stress-mediated autophagy signaling pathways, including PERK, eIF2α, ATF4, IRE1α, JNK, Beclin1 and ATF6 were significantly increased (*p* < 0.05), which were positively correlated with Cd concentration.

### 3.6 Effect of Cd exposure on endoplasmic reticulum autophagy and its relationship with endoplasmic reticulum stress

**Figure 5:**
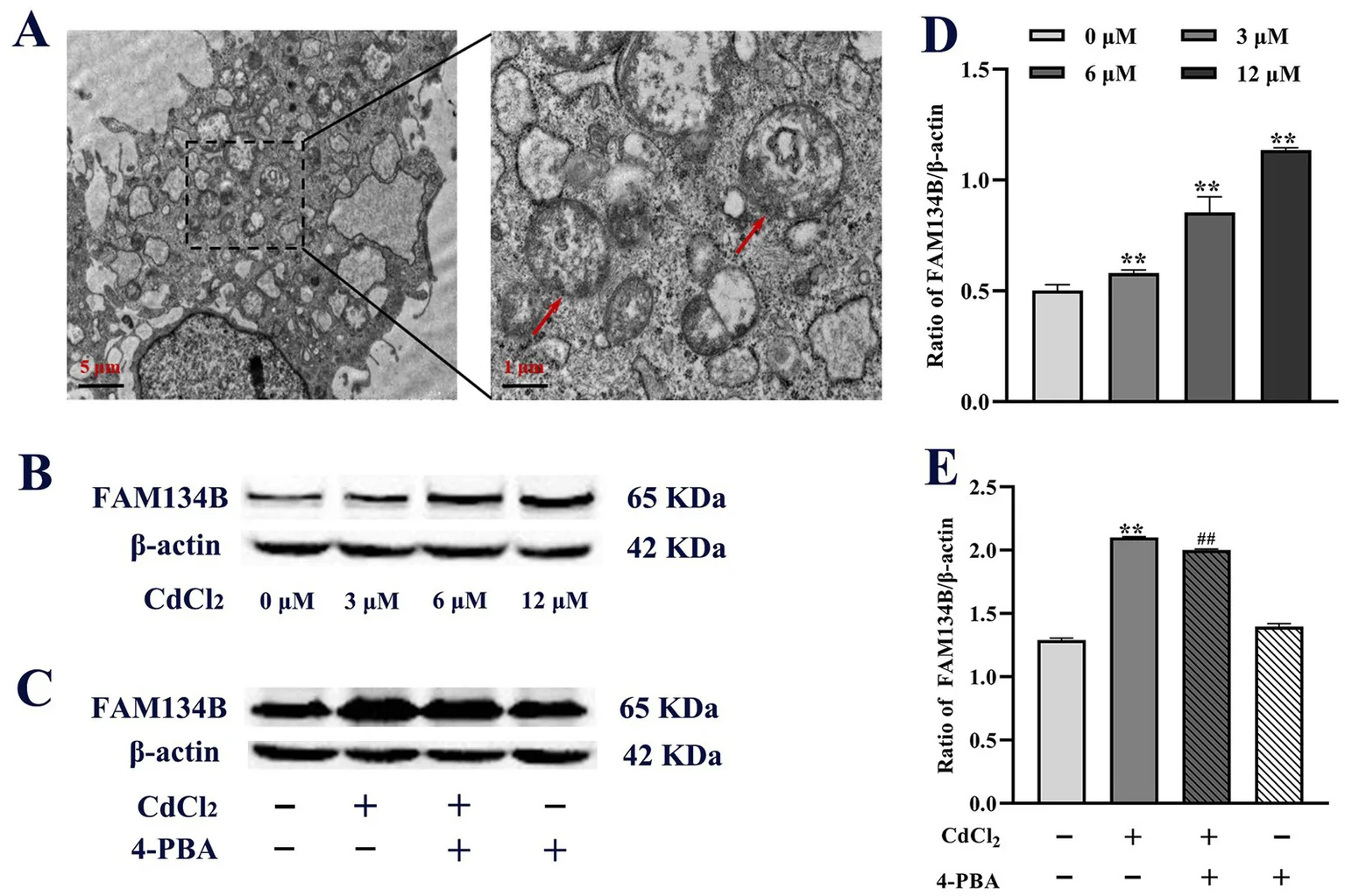
Effect of Cd exposure on ER-phagy and changes in ER-phagy after inhibition of ERS. **(A)** ER autophagosomes were observed under transmission electron microscopy. The red arrows refer to ER autophagosomes; **(B)** Western blot of FAM134B; **(C)** Western blot of FAM134B after inhibition of ERS; **(D)** Statistical chart of FAM134B expression; **(E)** Statistical chart of FAM134B expression after inhibition of ERS. The data are plotted using mean ± SEM, *n* = 6. **p < 0.01,*p < 0.05 indicate a significant difference compared to the control group. ^##^*p* < 0.01, ^#^*p* < 0.05 indicate a significant difference compared to the Cd group.

TEM analysis (Figure 5A) revealed that autophagosomes formed in Cd-treated BRL-3A cells enclosed folded vesicular membranes resembling ER-derived membranes, suggesting that Cd induces ER-phagy in hepatocytes.

Further detection of the expression of the ER-phagy receptor FAM134B (Figures 5B,D) showed that the expression of FAM134B was significantly increased in Cd-treated groups compared with the control, with the highest level observed at 12 μM Cd (*p* < 0.01). These results indicate that Cd exposure induces FAM134B-dependent ER-phagy.

To investigate the regulatory relationship between ERS and ER-phagy, pharmacological inhibition of ERS was performed using 4-PBA in Cd-exposed cells (Figures 5C,E). Western blot analysis revealed significant downregulation of FAM134B in the Cd + 4-PBA co-treatment group compared to Cd exposure (*p* < 0.01), demonstrating that Cd-induced ERS drives selective ER-phagy.

### 3.7 Effects of autophagy modulation on Cd-induced apoptosis

**Figure 6:**
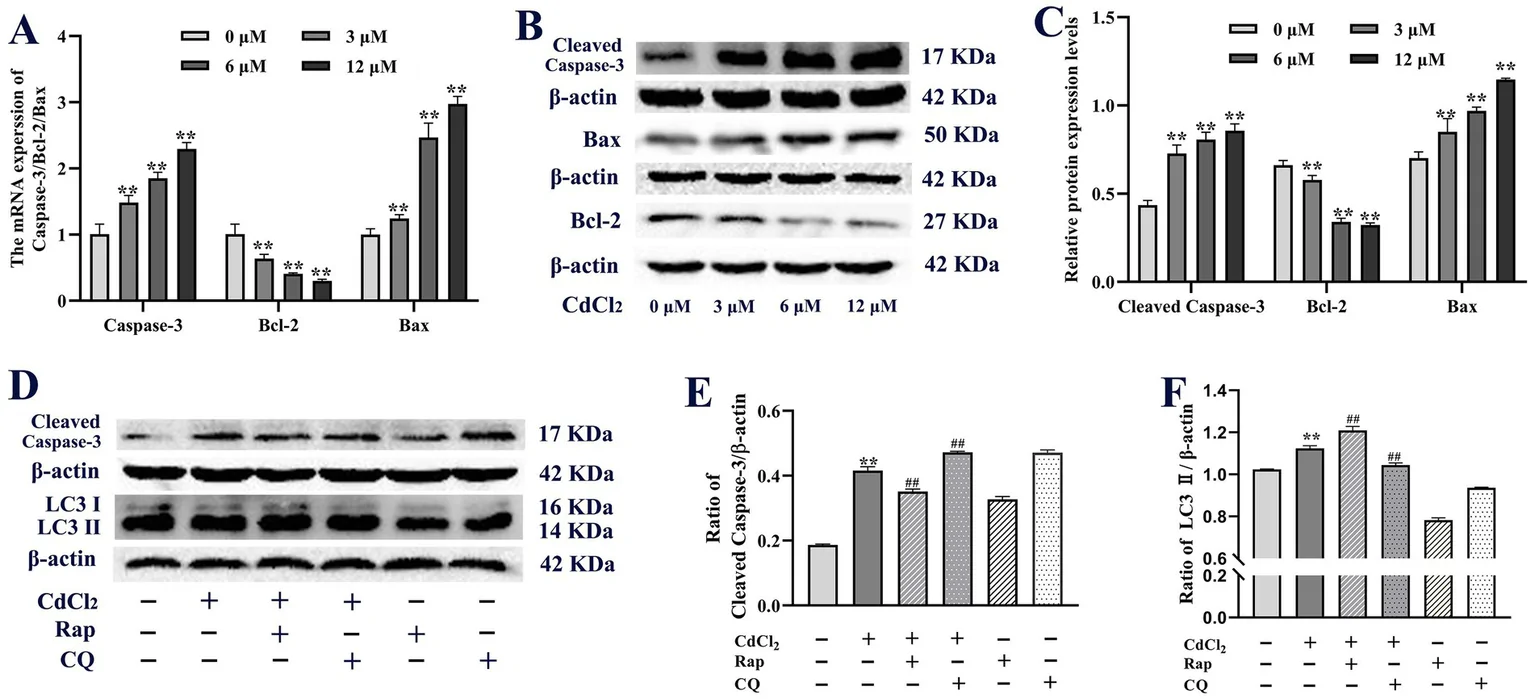
Role of ER stress-mediated autophagy in Cd-induced hepatocyte apoptosis. **(A)** mRNA expression of apoptosis markers; **(B)** Western blot of apoptosis markers; **(C)** Statistical chart of apoptosis markers expression; **(D)** Western blot of Cleaved Caspase-3 and LC3 after inhibition and activation of autophagy; **(E,F)** Statistical chart of Cleaved Caspase-3 and LC3II after inhibition and activation of autophagy. The data are plotted using mean ± SEM, *n* = 6; **p < 0.01,*p < 0.05 indicate a significant difference compared to the control group. ^##^*p* < 0.01, ^#^*p* < 0.05 indicate a significant difference compared to the Cd group.

We first examined the expression of apoptosis markers after treatment with 0–12 μM cadmium (Figures 6A–C). The results showed a dose-dependent increase in both mRNA and protein levels of the pro-apoptotic factors Cleaved Caspase-3 and Bax (*p* < 0.01), while the mRNA and protein expression of the anti-apoptotic factor Bcl-2 decreased in a dose-dependent manner (*p* < 0.01).

Further, we examined the relationship between autophagy and apoptosis during Cd exposure using Rap and CQ (Figures 6D–F). Compared to Cd-treated group, the Cd + Rap group exhibited increased LC3II levels (*p* < 0.01) accompanied by reduced Cleaved Caspase-3 expression (*p* < 0.01). Conversely, the Cd + CQ group showed decreased LC3II accumulation (*p* < 0.01) but elevated cleaved Caspase-3 expression (*p* < 0.01). These results demonstrate that Cd promotes apoptosis, and autophagy inhibition exacerbates Cd-induced hepatocyte apoptosis while autophagy activation exerts protective effects.

## 4 Discussion

Cd toxicity involves coordinated interactions among ERS, autophagy, and apoptosis, each exhibiting distinct functional roles. Previous studies have established the critical involvement of ERS and apoptosis in Cd-induced osteoblast dysfunction (27). Additionally, a study on Cd nephrotoxicity reported that Cd induced autophagy in duck renal tubular epithelial cells (28). Consistent with previous findings, our study demonstrates that Cd exposure triggers ERS, autophagy, and apoptosis in BRL-3A cells. Given the dynamic progression of autophagy, isolated morphological observations or single protein marker assessments often fail to comprehensively reflect autophagic activity. Therefore, we further examined the changes in autophagic flux, and our flux analysis revealed that the increase in autophagic markers was due not only to enhanced initiation but also to a blockade of degradative capacity. Similarly, one study reported that Cd exacerbates kidney damage by inhibiting autophagy in diabetic rats (29). In addition, Cd enhances the apoptosis of GC-2 cells by inhibiting autophagic flux in a mouse spermatocyte line GC-2 cell model (30). Although numerous studies have reported that Cd induces ERS and autophagy in cells, the regulatory relationship between these processes remains mechanistically undefined. Wang Xueru et al. (23) found that molybdenum and Cd-induced autophagy may be related to the antioxidant defense response in duck liver and the tandem activation between ERS. In the rat NRK-52E cell model, the regulatory relationship between ERS and autophagy was closely associated with Cd-induced nephrotoxicity (31). The data in this study showed that the autophagy-related proteins LC3II and P62 in Cd-treated BRL-3A cells were down-regulated when ERS was inhibited, suggesting that ERS acts as an upstream regulator of Cd-induced autophagy. Overall, our study found that Cd induces ERS activation, autophagic signaling, and apoptosis, while the increase in autophagosome accumulation is due to ERS-mediated autophagic initiation on the one hand, and Cd blocking autophagic degradation on the other.

Previous results have demonstrated that Cd-induced autophagic activation in BRL-3A cells is dependent on ERS. However, the molecular mechanism of autophagy activation in BRL-3A cells mediated by ERS under Cd exposure has not been reported. Under ERS conditions, the chaperone GRP78 dissociates from the ER transmembrane sensors IRE1α, PERK, and ATF6, each of which subsequently acts at distinct stages of autophagic signaling to induce autophagy (19). Activated PERK promotes the activation of eIF2α phosphorylation, which further regulates the activation of ATF4 (32). Autophosphorylation-activated IRE1α forms a complex with TRAF2 and ASK to promote the activation of JNK (33, 34). Activated JNK can promote the dissociation of Beclin1/Bcl-2 and release Beclin1 to initiate autophagosome nucleation (35, 36). In addition, activated IRE1α can promote the cleavage of XBP1 into XBP1 mRNA, and then transcriptionally activate Beclin1 to induce autophagic nucleation (37). ATF6 is transferred to the Golgi apparatus by vesicle transfer and is hydrolyzed and activated by S1P and S2P (38, 39). On the one hand, cleaved ATF6 can trigger autophagy by upregulating DAPK1 and then phosphorylating Beclin1 (40). On the other hand, ATF6 can directly upregulate the transcription of autophagy genes LC3, Atg3, Atg12 and Atg5 (41). Given that Cd induces ERS and autophagy, we examined whether these mechanisms are operative in BRL-3A cells. Consistent with this hypothesis, we found that Cd treatment increased protein and mRNA levels of PERK, eIF2α, ATF4, IRE1α, JNK, Beclin1, and ATF6 in BRL-3A cells in a dose-dependent manner. In summary, our study found that the mechanisms of autophagy mediated by ERS in response to Cd exposure include the PERK/eIF2α/ATF4, IRE1α/JNK/Beclin1, and ATF6 signaling pathways.

Bernales et al. (42) identified ER expansion and vesicle structures containing ER membranes by TEM in a yeast ERS model, naming them ER autophagosomes. Subsequent investigations identified FAM134B as the primary mammalian ER-phagy receptor and elucidated its role in selective autophagy (43). In the current study, TEM analysis of Cd-treated BRL-3A hepatocytes revealed autophagic vesicles encapsulating ER-derived membranes after 24 h exposure, prompting the hypothesis of Cd-induced ER-phagy. Supporting this, we observed dose-dependent upregulation of the ER-phagy receptor FAM134B following Cd exposure. In addition, given that Cd induces ER stress-mediated macroautophagy and ER-phagy in BRL-3A cells, we investigated their interplay. The data showed that inhibition of ERS significantly down-regulated the expression of FAM134B in the ER. These findings demonstrate that ER stress-mediated autophagy upon Cd exposure includes FAM134B-dependent ER-phagy.

The functional relationship between autophagy and apoptosis is context-dependent, with autophagy capable of either suppressing or promoting apoptotic cell death. In placental trophoblast models, autophagy has been shown to mitigate bisphenol A-induced apoptotic cell death (44). Conversely, research by Di et al. (45) revealed that autophagic activation facilitates Caspase-3 maturation through cathepsin D-mediated processing, thereby promoting apoptotic execution. The data from this study showed that rapamycin-induced autophagy activation decreased Cleaved Caspase-3 levels, whereas chloroquine-mediated autophagic inhibition elevated this apoptotic marker. These data indicate that ER stress-mediated autophagy serves a protective function against Cd-induced hepatocyte apoptosis.

In conclusion, this work delineates a molecular network associated with Cd-induced hepatotoxicity and identifies the ER stress-autophagy axis as a critical adaptive response against heavy metal insult. The finding that pharmacological enhancement of autophagy (via Rap) alleviates Cd-induced apoptosis highlights the therapeutic potential of targeting autophagic pathways. Modulating ER stress-mediated autophagy may represent a promising strategy for developing interventions to mitigate environmental Cd exposure-related liver diseases.

## Data Availability

The original contributions presented in the study are included in the article/Supplementary material, further inquiries can be directed to the corresponding author.
