# Supplementary material for: Role of endoplasmic reticulum stress-mediated autophagy in cadmium-induced apoptosis in BRL-3A cells
Source: Front Vet Sci. 2026 Aug 26;13:1939951. doi: 10.3389/fvets.2026.1939951 (PMC13553337; doi:10.3389/fvets.2026.1939951)
Supplement: Supplementary file 1 [file Supplementary_file_1.DOCX]

**Supplementary materials**

This supplemental material includes the **Supplemental methods**, **Supplemental Fig. S1**, **Table 1**, **Table 2**.

**Supplemental Methods**

**Cell Viability Assay**

The concentrations of the activator and inhibitor for the assay were selected by evaluating changes in cell viability after treatment with the drugs alone or in combination with Cd. Cell viability was assessed using the Cell Counting Kit-8 (CCK-8; BS350B, Biosharp, China). BRL-3A cells were seeded into 96-well plates at a density of approximately 2×10⁴ cells/well. After allowing the cells to adhere completely, the complete medium was replaced with medium containing varying concentrations of 4-PBA (0, 2.5, 5, 10, 20, 40, 80, and 100 mM) and incubated for 24 h. The cells were then washed twice with PBS, followed by the addition of 200 μL of fresh medium and 20 μL of CCK-8 solution to each well. After incubation at 37°C for 2 h, absorbance was measured at 450 nm using a BioTek Synergy H1 microplate reader.Next, the cells were treated with medium containing different concentrations of 4-PBA (0, 1, 2, 4, 8, 10, and 20 mM) along with 6 μM CdCl₂ for 24 h, and absorbance was measured again. Based on the calculated cell viability, the optimal concentration of 4-PBA was determined to be 10 mM.

Following the same protocol, cells were initially incubated with medium containing different concentrations of CQ (0, 5, 10, 20, 40, 60, 80, and 100 μM) for 24 h, and absorbance was measured after CCK-8 treatment. Subsequently, the cells were exposed to medium with varying concentrations of CQ (0, 10, 20, 30, 40, 50, 60, 70, and 80 μM) and 6 μM CdCl₂ for 24 h before measuring absorbance. From the cell viability results, the optimal CQ concentration was selected as 40 μM.

Similarly, cells were first treated with medium containing different concentrations of Rap (0, 10, 20, 40, 60, 80, 100, and 120 nM) for 24 h, followed by CCK-8 treatment and absorbance measurement. They were then incubated with medium containing Rap (0, 10, 20, 30, 40, 50, 60, 70, and 80 nM) and 6 μM CdCl₂ for 24 h before determining absorbance. Based on the cell viability data, the optimal Rap concentration was identified as 60 nM.

**Statistical Analysis**

All statistical analyses were performed using SPSS 26.0. Between-group comparisons were conducted via one-way ANOVA followed by least significant difference post-hoc tests. Results are expressed as mean ± SEM. Statistical significance was defined as follows: P > 0.05 (non-significant), P < 0.05 (significant), and P < 0.01 (highly significant).

**Supplemental figures**

B

A

C

D

F

E

**Fig. S1.** Screening of concentrations for 4-PBA, CQ, and Rap in the experiment. (A) Effects of different concentrations of 4-PBA (24 h) on cell viability; (B) Effects of co-treatment with different concentrations of 4-PBA and Cd (24 h) on cell viability; (C) Effects of different concentrations of CQ (24 h) on cell viability; (D) Effects of co-treatment with different concentrations of CQ and Cd (24 h) on cell viability; (E) Effects of different concentrations of Rap (24 h) on cell viability;(F) Effects of co-treatment with different concentrations of Rap and Cd (24 h) on cell viability. Data are presented as mean ± SEM (n=6). ******P* < 0.05 and *******P* < 0.01 indicate significant and highly significant differences compared to the control group **^#^***P*<0.05 and **^##^***P*<0.01 indicate significant and extremely significant differences compared to the Cd-treated group.

**Table 1**

Tab. 1 PCR primer sequence and parameters

| Genes | Primers used for PCR (5'-3') | Product length /bp |
| --- | --- | --- |
| *GRP78* | F:ATGGTGTGGGAGATCCTGTTTTC  R:CAAGACGCACAGGGATACGC | 124 |
| *CHOP* | F:CTGGGAAACAGCGCATGAAG  R:GTGGTCTCTACCTCCCTGGT | 121 |
| *Caspase-12* | F:TCGGAGAAGGAGCGAGCTTA  R:AGCTGTTTGTCGGAATTGGC | 121 |
| *LC3* | F:ACGGCTTCCTGTACATGGTC  R:GTGGGTGCCTACGTTCTGAT | 128 |
| *P62* | F: TATTACAGCCAGAGTCAAGG  R: CATCATACAGAAGCCAGAATG | 108 |
| *Caspase-3* | F:GGAGCTTGGAACGCGAAGAA  R:ACACAAGCCCATTTCAGGGT | 169 |
| *Bax* | F:CACGTCTGCGGGGAGT  R:CATCCTCTCTGCTCGATCCTG | 189 |
| *Bcl-2* | F:AGCATGCGACCTCTGTTTGA  R:TCACTTGTGGCCCAGGTATG | 108 |
| *PERK* | F:ACAAGGCTGTCACTCAGGTG R:GCTAGGAGCCTTGGAGCAC | 121 |
| *eIF2α* | F:TTTCCGGGACAAGATGGCG  R:AAGTGTGGGGGTCCATTCAC | 87 |
| *ATF4* | F:TGTTGGCGGGGGACTTAATG  R:AAAGGCATCCTCCTTGCCG | 200 |
| *IRE1α* | F:GCGCAGGTGCAATGACATAC  R:CATGCAAACTTCCGTCCAGG | 98 |
| *JNK* | F:TTGATTTTGGACTGGCGAGGA  R:TTGTGTGCTCCCTCTCATCTAA | 104 |
| *Beclin1* | F: GGAGATGTTGGAGCAAATGAA  R: GTCGCATTGAAGACATTGGTT | 92 |
| *ATF6* | F:TCATGGACCAGGTGAAGACT  R:GGGGCTCCATATGTCTGACTC | 169 |
| *β-actin* | F:CACCCGCGAGTACAACCTT  R:TCATCCATGGCGAACTGGTG | 72 |

**Table 2**

Tab. 2 Dilution multiple of antibody for experiment

| Antibodies | Identifier | Sources | Dilution ratio |
| --- | --- | --- | --- |
| GRP78 | PA1815 | Boster | 1:2000 |
| CHOP | GADD153 | Abcom | 1:10000 |
| Caspase-12 | 5528-1-AP | ABclonal | 1:12000 |
| LC3 | ab48394 | Abcam | 1:2000 |
| p62 | PM045 | Mblbio | 1:3000 |
| Cleaved Caspase-3 | WL01992 | Wanleibio | 1:2000 |
| Bax | 60267-1-lg | Proteintech | 1:5000 |
| Bcl-2 | 26593-1-AP | Proteintech | 1:5000 |
| PERK | ab229912 | Proteintech | 1:2000 |
| eIF2α | ab169528 | Abcom | 1:5000 |
| ATF4 | A18687 | Proteintech | 1:3000 |
| IRE1α | 27528-1-AP | Proteintech | 1:3000 |
| JNK | WL01295 | Wanleibio | 1:2000 |
| Beclin 1 | 11306-1-AP | Proteintech | 1:5000 |
| ATF6 | 24169-1-AP | Proteintech | 1:3000 |
| Horseradish peroxidase-conjugated anti-rabbit antibody | HA1006 | HuaBio | 1:12000 |
| Horseradish peroxidase-conjugated anti-mouse antibody | HA1001 | HuaBio | 1:12000 |
